# Sequencing and analysis of a South Asian-Indian personal genome

**DOI:** 10.1186/1471-2164-13-440

**Published:** 2012-08-31

**Authors:** Ravi Gupta, Aakrosh Ratan, Changanamkandath Rajesh, Rong Chen, Hie Lim Kim, Richard Burhans, Webb Miller, Sam Santhosh, Ramana V Davuluri, Atul J Butte, Stephan C Schuster, Somasekar Seshagiri, George Thomas

**Affiliations:** 1 SciGenom Labs Pvt Ltd., Plot 43A, SDF 3rd Floor CSEZ, Kakkanad, Cochin, Kerala, 682037, India; 2 Center for Comparative Genomics and Bioinformatics, Pennsylvania State University, 310 Wartik Lab, University Park, , Pennsylvania, 16802, USA; 3, , Personalis, 1350 Willow Road, Suite 202, Menlo Park, CA, 94025, USA; 4 Center for Systems The Wistar Institute,, , Philadelphia, PA, 19104, USA; 5 Division of Systems Medicine, Department of Pediatrics, Stanford University School of Medicine, Stanford, CA, USA; 6 Singapore Centre on Environmental Life Sciences Engineering, Nanyang Technological University, 60 Nanyang Drive, SBS-01N-27, Singapore, Singapore , 637551; 7 Department of Molecular Biology, Genentech Inc, 1 DNA Way, South San Francisco, CA, 94080, USA

**Keywords:** Indian genome, Personal genomics, Whole genome sequencing

## Abstract

**Background:**

With over 1.3 billion people, India is estimated to contain three times more genetic diversity than does Europe. Next-generation sequencing technologies have facilitated the understanding of diversity by enabling whole genome sequencing at greater speed and lower cost. While genomes from people of European and Asian descent have been sequenced, only recently has a single male genome from the Indian subcontinent been published at sufficient depth and coverage. In this study we have sequenced and analyzed the genome of a South Asian Indian female (SAIF) from the Indian state of Kerala.

**Results:**

We identified over 3.4 million SNPs in this genome including over 89,873 private variations. Comparison of the SAIF genome with several published personal genomes revealed that this individual shared ~50% of the SNPs with each of these genomes. Analysis of the SAIF mitochondrial genome showed that it was closely related to the U1 haplogroup which has been previously observed in Kerala. We assessed the SAIF genome for SNPs with health and disease consequences and found that the individual was at a higher risk for multiple sclerosis and a few other diseases. In analyzing SNPs that modulate drug response, we found a variation that predicts a favorable response to metformin, a drug used to treat diabetes. SNPs predictive of adverse reaction to warfarin indicated that the SAIF individual is not at risk for bleeding if treated with typical doses of warfarin. In addition, we report the presence of several additional SNPs of medical relevance.

**Conclusions:**

This is the first study to report the complete whole genome sequence of a female from the state of Kerala in India. The availability of this complete genome and variants will further aid studies aimed at understanding genetic diversity, identifying clinically relevant changes and assessing disease burden in the Indian population.

## Background

Since the publication of the first human reference genome in 2001, sequencing technologies have rapidly evolved, leading to increased throughput and reduced cost. Currently, one can obtain a complete human genome in less than two weeks at a cost of USD ~5000 or less, whereas the human genome project took over a decade and USD ~3 billion to complete. This advance has paved the way for obtaining personal human genomes quickly and inexpensively. Comparison of personal genomes and select regions of the genomes against the reference genome has provided a comprehensive view of human genetic diversity [1]. Rapid advances in sequencing technologies have enabled the identification of rare disease risk alleles and facilitated the practice of personalized medicine when making treatment decisions, though such applications are at their infancy [2-8].

Currently, published personal genomes predominantly represent individuals of European ancestry [9-13]. Genomes of individuals representing the Yoruba West-African, Han Chinese, South Korean, Khoisan and Bantu of Africa, Japanese, and Australian aborigines have also been published [14-19]. Recently, an Indian male genome was also published [20]. While a few studies have been conducted to understand the genetic diversity across populations in India, none have catalogued genetic variation at the whole genome level of a female individual from the subcontinent [20-22]. Understanding the extent of variations in the Indian population will be important for identifying clinically relevant changes in the Asian Indian subcontinent context.

Using a massively parallel sequencing approach, we have obtained the complete sequence of a South Asian Indian female (SAIF) genome. We identified over 3.4 million SNPs from this genome of which over 89,000 were found to be private SNPs. In performing an analysis of clinically relevant variants we have identified SNPs that indicate susceptibility to multiple sclerosis.

## Results

### Genome sequencing and alignment to the human reference

**Figure 1:**
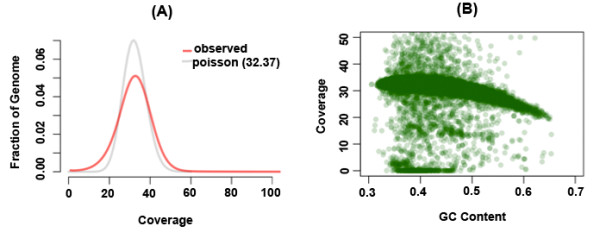
**Coverage characteristics for the sequenced genome.** The coverage calculations ignore potential PCR duplicates and secondary alignments. (**A**) Coverage depth distribution of the sequenced genome. The y-axis indicates the fraction of the non-N bases in the reference sequence that has a particular coverage on the x-axis. The curve in gray shows the Poisson distribution with the best fit to the distribution. (**B**) GC characteristics of the aligned data. We calculate and plot the GC percentage and average depth in non-overlapping windows of 50 kbp.

We generated 113.16 Gb of sequence data (1,131.56 million paired-end reads of length 100 bp) that was aligned to the human reference sequence (GRCh37/hg19; 2,861,343,702 non-N bases) using BWA [23]. We aligned 96.27% of the reads (99.97 Gb) to the reference sequence resulting in an average coverage of 34.94 fold across the genome (Table 1). The coverage depth distribution of the sequenced genome is shown in Figure 1A along with a Poisson distribution with the same mean value. Compared to the Poisson distribution, which has been used to model sequence data in several earlier studies, we observe that the coverage distribution has more weight on both tails. A decrease in the average coverage with increasing GC content in 50 Kb non-overlapping windows across the whole genome was also observed (Figure 1B). While 98.89% of the reference genome was covered by at least five reads (required for variant calls), 99.17% of it was covered by at least one read.

**Table 1:** Sequencing and analysis statistics

Total paired-end raw reads (each of 100 bases) in million	1,131.56
Total raw bases (Gb)	113.16
Total mapped bases (Gb)	99.97
Mean mapped depth (x)	34.94
Bases accessed (% of genome)	99.17
Total SNPs	3,459,784
Total Indels	384,926

We performed a *de novo* assembly of reads that did not align to the chromosomes in GRCh37, using SOAPdenovo [24]. This generated 57,426 contigs comprising 23,683,357 bases with an average contig length of 412 bp. Of these, 42.69% sequences aligned to the unanchored contigs and chromosomes in GRCh37 and another 9.25% of the sequences aligned to the alternative human assemblies. About 33.05% of the assembled sequences aligned to other human sequences in the NT database, while another 3.64% of the sequences aligned to non-human primates with an E < 10^-5^.

### SNPs and indels

**Figure 2:**
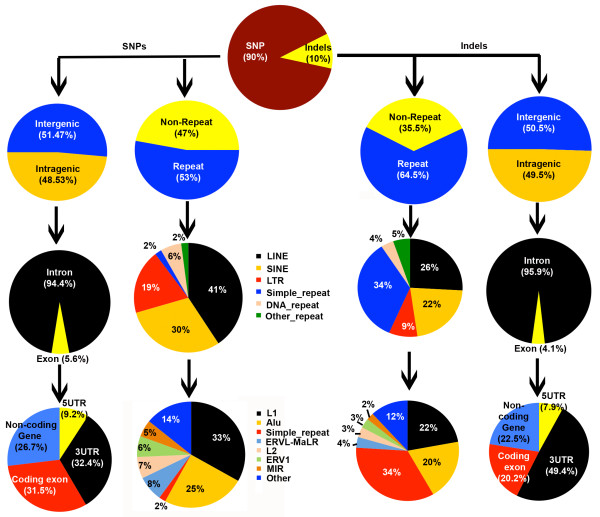
**Annotation of SNPs and Indels.** The SNPs and Indels coordinates were compared with gene boundary, exon boundary, untranslated and protein-coding regions, repeats, repeat classes and subclasses.

We identified a total of 3,459,784 single nucleotide variants (2,087,876 heterozygous) in this genome, by comparing it against human reference genome assembly (GRCh37, also known as hg19), using methods previously described [14]. The SNP calls were further validated using Illumina HumanOmni1-Quad BeadchipSNP array data. We observed a 98.7% concordance between the SNP calls made using the sequencing data and the SNP array, confirming the validity of the sequencing derived SNP calls. The single nucleotide variants identified in the SAIF genome are referred to as SNPs (relative to the human reference genome) in the results below and this does not include single base insertions/deletions. Of the total SNPs identified, 1,679,111 (48.5%) mapped to gene (intragenic) coding regions of the genome. Given that exons form a small part of the gene coding region, of the total intragenic SNPs identified, only 5.6% (94,247; 29,724 in coding exons, 25,354 in non-coding exons, 8,651 in 5’UTR and 30,518 in 3’UTR) mapped to them (Additional file 1: Table S1A). This is consistent with the lower mutation rates typically observed with coding regions of the genomes [25]. Among the SNPs in coding exons, 11,155 are synonymous (syn) substitution that are distributed among 6,631 genes and 11,216 are non-synonymous (non-syn) changes that map to 6,279 genes. This is consistent with a non-syn:syn (dN/dS) ratio of ~1 expected of a normal genome [26]. Of the SNPs identified, 1,832,801 (53%) mapped to repeat containing regions of the genome [27,28]. Further, we found that about two-thirds of the SNPs identified in the repeat regions were found in long interspersed elements (LINE; 41%; majority occurring in L1 elements) or short interspersed elements (SINE; 30%; majority occurring in Alu elements; Figure 2, Additional file 1: Table S1B).

In addition to SNPs, insertions and deletions (indels) are a class of variations that shape evolution of genomes [29,30]. In the SAIF genome, of the total 384,926 indels identified, 190,533 (49.5%) were found in gene coding regions. As observed with SNPs, only 7,871 (4.1%) of indels (1,591 in coding exons, 1,769 in non-coding exons, 620 in 5’UTR and 3,891 in 3’UTR) occurred within exons. Of the total indels, 248,309 (64.5%) were found in repetitive regions, proportionally higher than SNPs that occurred in this region. This very likely reflects the slippage that occurs during replication leading to increased occurrence of indels in repeat regions [31]. Further, it is interesting to note that while indels were predominant (34%; 85,193) in simple repeats (Figure 2, Additional file 1: Table S1B), only 2% of the SNPs were found in the simple repeat regions.

**Figure 3:**
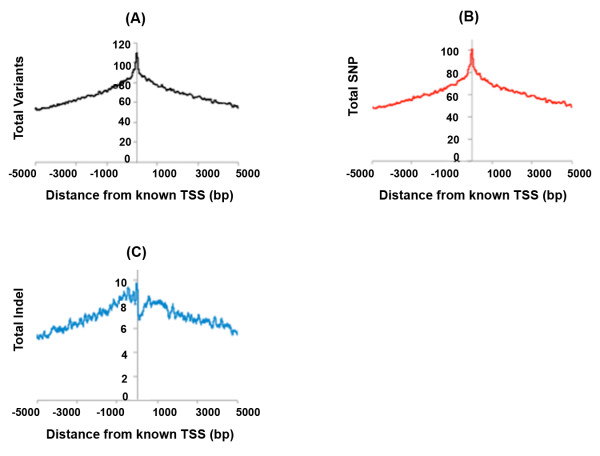
**Average enrichment of variant around TSS of genes.** (**A**) Average enrichment of all variants (SNPs + Indels) around TSS, (**B**) Average enrichment of SNPs around TSS, (**C**) Average enrichment of Indels around TSS.

The presence of SNPs and indels can affect the gene regulatory regions such as transcription factor binding region (TFBR)/promoters and enhancer sites. We first looked at the average enrichment of SNPs and indels around transcriptional start sites (TSS) of known genes. Interestingly, we observed an increased SNPs density around TSS, suggesting an important role for variations in modulating expression across individuals [32]. In contrast to SNPs, there were fewer indels downstream of TSS (around 50 bp downstream; Figure 3). This likely suggests a need for preserving the promoter length/architecture around TSS. We further investigated all conserved TFBR and enhancer sites catalogued by UCSC genome browser [33] and VISTA enhancer browser [34], respectively in the SAIF genome to understand the extent of variations in these regions. For TFBR, we focused on SNPs and indels that are present within 5 kb upstream of the genes. We found 1,328 SNPs and 66 indels within the TFBR and 1,732 of SNPs and 203 indels in the enhancer sites. The top TFBR SNP containing sites included regions adjacent to Forkhead box J2 transcriptional activator protein, myocyte enhancer factor-2 involved in cellular differentiation, Brachyury protein involved in mesoderm formation and differentiation, CHX10 protein involved in progenitor cell proliferation and bipolar cell determination in developing retina, and the peroxisome proliferator-activated receptor-gamma (PPAR-gamma) protein that regulates adipocyte differentiation. In general, we found genes involved in cancer pathways to be enriched for SNPs in their promoter regions (FDR < = 0.05).

### Coding SNPs are predominant in olfactory genes

**Figure 4:**
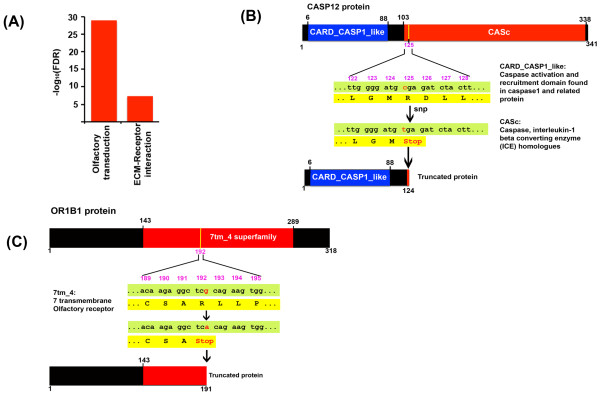
**(A) Pathway enrichment analysis.** The enriched KEGG pathways (FDR < = 0.05) identified are reported. (**B**) Non-sense SNP (C > T) in codon 125 of CASP12 leads to its truncation. (**C**) A variant in OR1B1 gene results in a premature stop and truncation of OR1B1.

Coding SNPs (cSNPs) can lead to amino-acid substitutions in proteins encoded by the genome. In the SAIF genome, of the 22,371 cSNPs that occur in the protein coding sequence 11,216 lead to non-synonymous (nsSNP) substitutions (Table 2). While the synonymous SNPs (sSNPs) map to 6,631 genes, the nsSNPs are distributed among 6,279 protein-coding genes in the genome. To understand the significance of the cSNPs, we assessed their enrichment in KEGG pathways using DAVID [35]. While distribution of both sSNPs and nsSNPs was significantly enriched (FDR < = 0.05) in ECM-receptor interaction pathway genes, only the olfactory transduction pathway genes showed a statistically significant enrichment for nsSNPs (Figure 4A, Additional file 2: Figure S1). This is consistent with the higher levels of polymorphism observed in human olfactory gene family [36]. The nsSNPs included 11,107 missense substitutions and 109 non-sense mutations that lead to premature stop (Additional file 1: Table S2). Genes with premature stop included *CASP12,* a cysteine protease involved in inflammation and innate immune response, and *OR1B1*, an olfactory receptor interacting with odorant molecules in the nose. The CASP12 protein contains an N-terminal caspase activation and recruitment domain (CARD) and a C-terminal catalytic cysteine protease domain characteristic of caspase family of proteins (Figure 4B). The non-sense mutation observed in the SAIF genome codes for a truncated CASP12 protein that contains just the N-terminal CARD domain. Recent re-sequencing studies have shown that this truncated form of CASP12 confers resistance to sepsis and is predominant across many populations [37-39]. Another non-sense mutation in the SAIF occurred in the *OR1B1*, which encodes a G-protein coupled olfactory receptor. The OR1B1 SNP leads to truncation of the 7-transmembrane receptor domain present in OR1B1 (Figure 4C). This truncated variant of OR1B1 protein has been observed in a recent study [39] and is thought to affect metabolism of serum cholinesterase [40]. Overall, our assessment of the effect of the nsSNPs using SIFT [41] indicates that 1,460 are likely damaging (Additional file 1: Table S3).

**Table 2:** Variants with Gene coding regions

**Class**	**Total**
Synonymous SNPs	11,155
Non-synonymous SNPs	
Missense type	11,107
Non-sense type	109
Coding-region Indels	
In-frame Indels	172
Frame-shifting Indels	200

Unlike SNPs, indels in coding regions, in addition to non-sense mutations, can lead to frame shift changes. Of the 372 coding region indels, 172 are in-frame and 200 lead to frame-shift change (Additional file 1: Table S4A, Additional file 1: Table S4B). Genes where the indel leads to a frame-shift includes HIF3A, hypoxia inducible factor 3 alpha subunit, thought to be a negative regulator of hypoxia-inducible gene expression; MMP28, a matrix metallopeptidase involved in the breakdown of extracellular matrix for both normal physiological and disease processes; and HNF1A, a transcription factor required for the expression of several liver-specific genes. The frame-shift at position 147 in MMP28 protein introduces a premature stop codon at 179. This results in loss of zinc-dependent metalloprotease and hemopexin-like repeat domain, leading to a truncated MMP28 protein that lacks a catalytic domain (Additional file 2: Figure S2). SIFT analysis of the indels indicated 126 indels to be deleterious (Additional file 1: Table S5).

### Comparison and novel variants

We compared SAIF SNPs against those from other published personal genomes, the variations from the 1000 Genomes Project and dbSNP database (dbSNP132). The personal genomes used to perform the comparison had a sequencing coverage of at least 10X. Shared SNP sites, where both the SAIF genome and the genome it is compared to carry a SNP, provide a measure of the degree of similarity between the genomes. We also compared the indels found in the SAIF genome with those reported by the 1000 Genomes Project.

SNPs level comparison of the SAIF genome found that this individual shared 48.77% of the SNP sites with NA12891 (Caucasian) genome, 48.82% with the NA12892 (Caucasian) genome, 52.5% with the Venter (Caucasian) genome, 50.68% with the NA18507 (YRI) genome, 44.29% with the NA19238 (YRI) genome, 44.33% with NA19239 (YRI), 53.75% with YH (Han Chinese) genome, 59.24% with SJK (Korean) genome, 46.5% with ABT (South Africa) genome, 51.1% with Irish (Caucasian) genome, 49.86% with KB1 (Southern Kalahari, Africa), 59.41% with the recently published Indian male genome [20], 95.18% with dbSNP 132, and 92.44% with 1000 Genomes Project variation collection.

**Figure 5:**
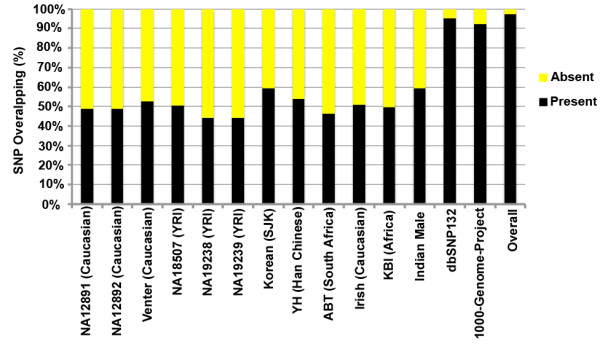
Comparison of SAIF SNPs with 12 published personal genomes, 1000 Genomes Project SNPs and dbSNP132.

Overall, we found that 2.6% (89,873) of the SNPs and 83.83% (322,295) of the indels to be unique to SAIF genome (Figure 5, Additional file 1: Table S6). The complete list of novel SNPs and indels is provided in Additional file 1: Table S7. Of the novel SNPs, 22,412 (24.94%) mapped uniquely to genes, 28,313 (31.5%) mapped specifically to repeat regions, 21,826 (24.29%) mapped to both gene coding and repetitive regions and 17,322 (19.27%) mapped to other regions of the genome. Further, of the total novel SNPs that mapped to coding regions, 543 led to protein level alterations (533 missense SNPs and 10 non-sense substitutions). Genes having novel non-sense substitutions include TSG101, a phosphoprotein implicated in tumorigenesis and the CD164 gene that plays a role in hematopoiesis. Further, assessment of the protein altering novel SNPs using SIFT predicted 154 of these to be likely damaging.

### mtDNA analysis

**Figure 6:**
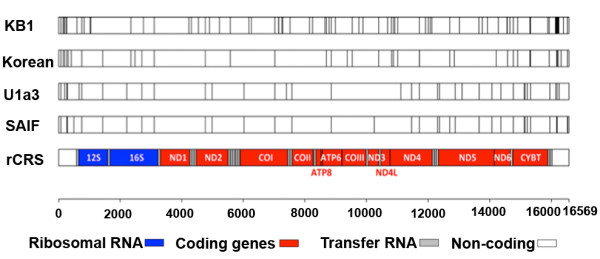
**Graphical representation of SAIF mitochondrial (mt) genome in comparison to Cambridge reference sequence (rCRS).** Vertical bar in the individual mt genomes indicates SNP positions in comparison to rCRS. The SAIF mt DNA has 35 SNPs compared to rCRS. The closest haplotype of the SAIF sequence, the U1a3 Haplotype (AY714038) has 37 SNPS compared to rCRS. The Korean mt DNA [16] has 40 SNPs and the KB1 southern African [14] had 80 SNPs in comparison to rCRS.

Mitochondrial DNA (mtDNA) sequence is widely used to understand the maternal genetic history of human populations [42]. Analysis of the SAIF mt genome showed that it had 35 SNPs (control region 9, non-coding region 1, RNA genes 5 and protein coding genes 20; syn/non-syn ratio of 16/4) compared to the Cambridge reference sequence (rCRS, Figure 6). The closest mtDNA sequence of SAIF among the mtDNA in GenBank was AY714038, belonging to U1a3 haplogroup (Additional file 2: Figure S3). This sequence was reported from a study involving the Indian population [43] and contained 14 nucleotide differences compared to the SAIF mt genome. Therefore, the most related haplogroup with the SAIF mt genome was the U1a3 haplogroup. This is consistent with the fact that the SAIF individual is from the southern Indian state of Kerala where the frequent occurrence of U1 haplogroup has been previously reported [44]. The coalescence time for the U1a lineage was estimated to be about 46 kya (Additional file 2: Figure S4). This deep divergence and genetic distance between SAIF and its closely related haplogroups suggest that the SAIF mt genome belongs to one of the distinctive lineages within the U1a haplogroups.

### SNPs with health and medical relevance

We assessed cSNPs identified in the SAIF genome using annotations in SNPedia and OMIM for their health and disease relevance. This analysis identified 59 and 63 cSNPs with implications in health and disease from SNPedia and OMIM databases [45,46], respectively (Additional file 1: Table S8 and Additional file 1: Table S9). Interestingly, this analysis revealed several SNPs with implications for susceptibility to cancer and cardiovascular diseases. The cancer susceptibility SNPs included the variation in SDHB gene (S163P, OMIM_ID #185470.0015), responsible for Cowden-like syndrome, resulting in enrichment of carcinomas of human breast due to downstream inactivation of PTEN [47]. We also found an exon 10 BRCA2 variant (N372H; OMIM_ID # 600185.0133), [48] and an EPCAM variant identified in Chinese population (M143T; rs1126497; [49]) that are associated with increased risk for breast cancer. Further, a SNP in CENPF gene (R2943G; rs438034) that occurs in the SAIF genome is associated with a poor breast cancer survival [50]. Other SNPs with increased cancer susceptibility include FCGR2A H166R (rs1801274) associated with increased risk for non-Hodgkin’s lymphoma [51], ANKK1 E713K (rs1800497; [52]) involved in advanced adenoma recurrence, HNF1A S487N (rs2464196; [53]), MMP9 Q166R (rs17576-rs2250889; [54]), and XPC Q939K (rs2228001; [55]) variants associated with lung cancer, ATG16L1 T137A (rs2241880; [56,57]) with Crohn’s disease, and OGG1 P332A (rs1052133; [58-60]) associated with bladder and gall-bladder cancer in Japanese, Chinese and Indian populations. An ATR (M211T; rs2227928) variant found in the genome has been associated with a poorer response to gemcitabine and radiation therapy in pancreatic cancer [61]. We also found a protective SNP that occurs in the PON1 gene (Q192R; rs662) that is known to lower (0.65x) risk for ovarian cancer [62]. Two common missense variations in ELAC2 gene (A541T; OMIM_ID # 605367.0002 and S217L; OMIM_ID # 605367.0001) implicated in genetic susceptibility to heredity prostate cancer were found in the SAIF genome. This while not of direct significance to SAIF individual, could be of relevance to the male children, if any [63-65].

The cardiovascular disease associated SNPs found in this individual include those in LRP8 (R952Q; rs5174/OMIM_ID # 602600.0001; [66]) and MMP9 (Q166R; rs17576; [67]) both of which increase risk for myocardial infarction, ROS1 (S2229C; rs619203; [68]) variation associated with increased coronary heart disease, AKAP10 SNP (I646V; OMIM_ID # 604694.0001; [69]) associated with cardiac conductivity defect susceptibility and ADRB3 variant (W64R; rs4994; [70]) implicated in higher risk of cardiac events. Also, two SNPs in the PON1 (Q192R; rs662 and L55M; rs854560) show a high risk of cardiovascular disease [71] and a higher risk of coronary artery disease [72,73]. A SNP in SNX19 (L878R; rs2298566) is linked to elevated risk of coronary heart disease but has also been shown to be associated with better response to statins and may be of clinical significance [74]. Other SNPs affecting cholesterol levels (EDN1 K198N; OMIM_ID # 131240), familial obesity (FAM71F1 E143K; rs6971091) and hypertension susceptibility (PPARFC1A, G482S; rs8192678 and CYP4A11, F434S; rs1126742) were also found in the genome.

In addition to this, several other SNPs associated with Alzheimer’s disease, diabetes, tuberculosis susceptibility and macular degeneration were also detected. A SNP in ICAM1 (K469E; rs5498), associated with increased resistance to malarial infection, originally identified in a study of over 552 Indian individuals [75], was also observed in the SAIF genome. It must be noted that a majority of the SNPs of health relevance used to annotate the coding SNPs were derived from studies involving western populations. Hence, validating the relevance of these in the context of Asian Indian population will require controlled studies in a cohort representative of the Indian subcontinent.

**Figure 7:**
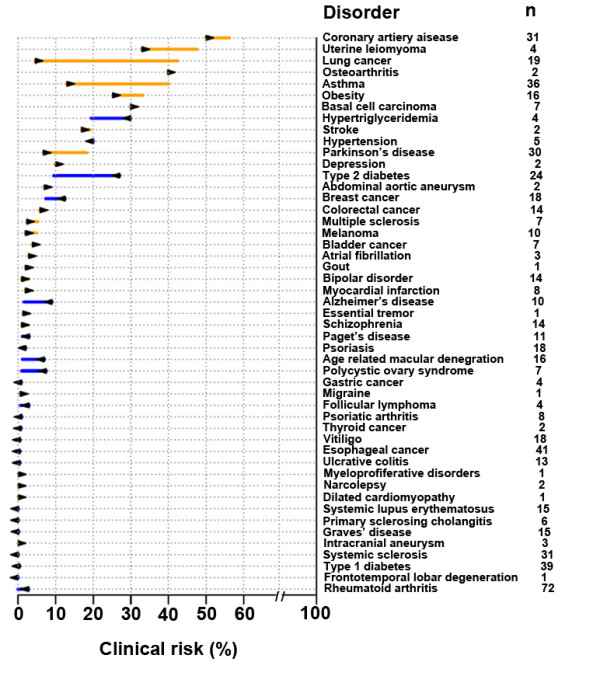
**RiskOgram for 49 diseases.** For each disease, the arrow represents the pretest probability according to the subject’s age, gender, and ethnicity. The line represents the post-test probability after incorporating the subject’s genome sequence. Orange line represents increased genetic risk, while blue line represents decreased genetic risk. Listed to the right are the numbers of independent disease-associated SNPs used to calculate the subject’s post-test probability.

Besides assessing the cSNPs using SNPedia and OMIM, we performed a comprehensive assessment of predicted genetic risk of the SAIF genome for 49 diseases using Varimed [76]. As described recently [77], we first estimated the pre-test probability using the prevalence of each disease according to the age, gender, and ethnicity of SAIF. Using this analysis of the SAIF genome we found 17 diseases that had post-test probability >5% (Figure 7).

We further assessed the relative genetic risk of SAIF against Gujarati Indians in Houston (GIH) population represented in HapMap III. We used the set of disease SNPs measured in both SAIF and GIH, and recalculated the likelihood ratio (LR) for SAIF and each of 101 GIH individuals. We found that the SAIF individual had a higher genetic risk than 80% of GIH for eight diseases (Additional file 2: Figure S5). Intersecting both results, we found that SAIF had a high genetic risk for four diseases, including multiple sclerosis (post-test probability = 5%, relative risk > 100% GIH), uterine leiomyoma (post-test probability = 47%, relative risk > 97% GIH), asthma (post-test probability = 17%, relative risk > 90% GIH), and obesity (post-test probability = 34%, relative risk > 82% GIH).

**Figure 8:**
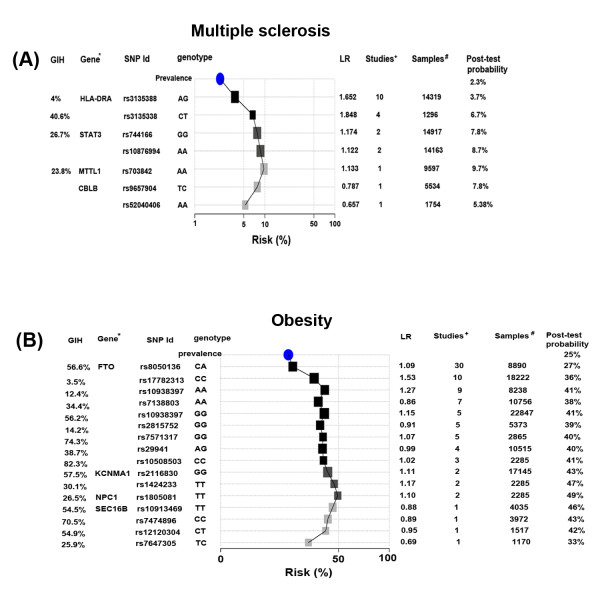
**Contribution of individual SNPs to overall risk for (A) multiple sclerosis and (B) obesity.** Single nucleotide polymorphisms (SNPs) with associations established from genome-wide association shown in decreasing order of number of studies showing association for a given SNP. An increasing shade of black filling squares corresponding to each SNP on the graph is indicative of the number of studies that reports a disease association for that SNP. The size of boxes is proportional to the logarithm of the number of samples used to calculate the likelihood ratio (LR). The post-test probabilities were computed using the pre-test estimate as a starting point. An updated combined post-test risk probability is shown along the y-axis that includes the contribution of indicated SNP and those above it on the graph. *Gene names are shown where the SNP lies in an annotated gene region. +Number of studies reporting an association. #Number of samples used to calculate the LR. GIH- The frequency of a SNP in the Gujarati Indian Population from the Varimed database is also shown.

SAIF’s high genetic risk on multiple sclerosis is based on a rare heterozygous AG variant at rs3135388 in HLA-DRA (Figure 8), which appears in 4% GIH individuals. Multiple studies have linked the presence of the “A” allele with an increased risk for multiple sclerosis in American, Australian, British, Canadian, Dutch, mixed European, and Serbian populations [78-87]. International Multiple Sclerosis Genetics Consortium used this allele as a proxy for the DRB1*1501 allele, which had been demonstrated as a causal variant for multiple sclerosis and validated in animal models in OMIM (OMIM_ID #126200). This variant has also been validated as a functional regulatory variant, with evidence from transcription factor binding site, eQTL, and DNase peak from Regulome DB ( http://www.regulomedb.org/) with data from ENCODE.

In addition to multiple sclerosis, SAIF had a high genetic risk of uterine leiomyoma, driven by a rare heterozygous CT variant at rs7913069 (Additional file 2: Figure S6). The T allele had been validated to increase the risk of uterine leiomyoma with an odds ratio of 1.47 and p-value = 8.65 × 10^-14^ in Japanese women [88]. A high genetic risk for asthma and obesity were also identified in the SAIF individual [89,90] (Figure 8, Additional file 2: Figures S7 and Additional file 2: Figures S8).

### SNPs of pharmacogenomic relevance

An individual’s SNPs can be used to predict adverse drug reactions and also manage the dose of drugs prescribed. In order to understand SNPs of pharmacogenomic relevance, we evaluated SAIF variants using annotations present in the PharmGKB database [91] and a set of published SNPs relevant for drug interactions. We identified 109 SNPs with pharmacogenomic relevance based on PharmGKB (Additional file 1: Table S10). We identified 10 actionable SNPs from literature as it pertains to treatment with various drugs, some of which are also represented in the PharmGKB (Table 3). As India has a high burden of diabetes, we looked at the SAIF genome for a SNP in ATM previously associated with metformin response [92]. Metformin is a drug widely prescribed to manage blood sugar levels in diabetics. We found a GG (rs4585) variant in ATM in the SAIF genome and this is associated with positive response to metformin [92]. FDA in the US has recommended testing for SNPs that help in deciding the dosage of warfarin, used as a blood thinner. Based on the three SNPs (Table 3) found in the SAIF genome, we conclude that a typical dose of warfarin is not likely to cause bleeding. Given that the SAIF genome SNPs predicted an increased risk for multiple sclerosis, we looked at genes with SNPs that predict response to therapy in the context of multiple sclerosis. We found a CT variant (rs7987675) in GPC5, which is associated with typical response to beta interferon therapy in relapsed multiple sclerosis patients. Besides these expected drug responses, we have identified additional ones based on the SAIF genome and they are detailed in Table 3.

**Table 3:** Drug response SNPs

**#**	**Drug/Condition**	**Chromosomal location**	**Gene**	**Genotype**	**SNP-ID**	**Drug related outcome**
1	Interferon beta therapy for multiple sclerosis (MS)	chr13:92884370	GPC5	CT	rs7987675	will likely not show increase response to interferon beta therapy in case of relapsed MS
2	Lumiracoxib-related liver toxicity	chr6:32305978	C6orf10	GT	rs3129900	increase in liver toxicity risk in response to lumiracoxib used to treat acute pain and osteoarthritic symptoms
3	Metformin Response	chr11:108239628	ATM-C11orf65	GG	rs4585	will respond better to Metformin
4	Ribavirin-induced anemia	chr20:3193842	ITPA	CA	rs1127354	greatly decreased odds of developing anemia when taking PEG-IFN/RBV
5	Statin induced myopathy	chr12:21331549	SLCO1B1	TT	rs4149056	typical dose of Simvastin will not increase myopathy risk
6	Floxacillin and liver toxicity	chr6:31431780	HCP5	TT	rs2395029	at typical dose liver toxicity is not expected in response to floxacillin
7	Beta-Blocker - heart failure risk	chr10:115805056	ADRB1	CC	rs1801253	Bucindolol is unlikely to reduce mortality odds in case of heart failure
8	Response to amitriptyline	chr7:87160561	ABCB1	AA	rs2032583	typical response to depression when treated with Elavil, Paxil, Effexor, or Celexa
9	Warfarin sensitivity	chr10:96702047	CYP2C9	CC	rs1799853	typical dose of warfarin does not increase risk of bleeding
		chr10:96741053	CYP2C9	AA	rs1057910	
		chr16:31107689	VKORC1	CC	rs9923231	
			promoter			
10	Fluorouracil toxicity	chr1:97915613	DPYD	CC	rs3918290	No copies of the DPYD*2A mutation. May still be at risk for 5-FU toxicity due to other genetic or non-genetic factors

## Discussion

We have sequenced the genome of a female from Kerala in southern India and identified 3,459,784 SNPs and 384,926 short indels. Comparison with published personal genomes revealed that SAIF shared ~50% of the SNPs with each of the personal genomes published so far and had 89,873 private SNPs. Of the total SNPs detected, we identified 11,107 missense substitutions and 109 non-sense mutations. We found olfactory genes to be enriched for non-synonymous SNPs suggesting that this family of genes may be under reduced evolutionary constraint in humans. Besides the nuclear genome, analysis of the mitochondrial genome showed that SAIF mitochondria belonged to the U1 haplogroup which is known to occur in the southern Indian state of Kerala.

SNPs in personal genomes can be used to assess disease risk, carrier status and drug response/interaction. We have assessed the SAIF genome using OMIM, SNPedia and Varimed databases for SNPs with health and disease consequences. We identified higher risk for multiple sclerosis, among other diseases. Drug response related SNP assessment revealed that the SAIF genome carried a SNP in the ATM gene that predicts a favorable response to metformin used in treating diabetes. These and the other annotations made using experimentally verified variants will very likely be used by physicians for counseling and making treatment decisions.

A recent study on variations in India using SNP array suggest that genetic diversity within India is at least three times that observed within Europe [22]. In India, burden of recessive genetic disorders is predicted to be high and likely to be unique within each population group [93]. Additional personal genomes from Indian subcontinent that represent population groups within India will be critical to assessing the variation and disease burden.

## Conclusions

In this study we report the first complete sequence of a south Asian Indian female from the state of Kerala in India. The availability of this genome and the variants identified is a first step in understanding the genetic diversity in the Indian subcontinent. In addition, the clinically relevant changes identified in this personal genome, along with further studies on additional genomes from this region, should provide a comprehensive assessment of the disease burden in the Indian population.

## Methods

### Sample collection, library construction and sequencing

Informed consent was obtained from the individual prior to initiation of this study. The donor is a healthy 48 year old female from Kerala in the southern part of India. Blood sample (8.5 ml) was collected in a PAXgene Blood DNA Tube (Qiagen, CA) and processed as per manufacturer’s instructions. High molecular weight genomic DNA obtained was sheared and used in the preparation of the whole genome shotgun libraries as per Illumina’s library preparation protocols (Illumina, CA). The libraries were then sequenced on a HiSeq 2000 sequencing machine (Illumina, CA) to obtain the sequence data.

### Alignment to the reference

We used BWA (version 0.5.9) to align the reads to the human reference sequence (GRCh37/hg19). All default parameters were used, with the exception of “-q 15” which allows read trimming at the 3’ ends, down to 35 bp, prior to alignment. Potential PCR duplicates, which can adversely affect the variant calls, were removed using the MarkDuplicates tool from Picard version 1.4.0 ( http://picard.sourceforge.net). The resulting BAM file was used for all subsequent analysis.

### *De novo* assembly of unaligned reads

We used SOAPdenovo with a *K*-mer size of 39 and with the “-R” option to use reads to solve tiny repeats. The resulting contigs were first aligned to unanchored contigs in hg19 using LASTZ requiring an identity > 95% and requiring more than 80% of the assembled contig sequence in the alignment. The reads that did not align to hg19 were compared using BLAST [94] against all existing human assemblies using BLASTN requiring an E < 10^-5^. The remaining reads were then analyzed using BLAST against the NT database.

### SNP and Indel identification

We used SAMtools (version 0.1.12a) to call variants (substitutions and small indels) from the alignments generated above. All default parameters were used in conjunction with “-C 50” to reduce the effects of the sequences with excessive mismatches. The variants were filtered to keep the ones where the depth of coverage was > = 5 and < = 60 for all chromosomes except the mitochondria. A total of 3,620,895 single nucleotide substitutions and 509,994 indels were identified in this sample, and we further filtered the variants to only keep the ones with a SNP quality score > = 30. Also, heterozygous variants that did not share any alleles with the reference sequence were excluded. The SNP calls made using the whole genome sequencing data were further validated using SNP calls for this individual derived using Illumina HumanOmni1-Quad Beadchip SNP array. We found that the calls between sequencing data and the SNP to be concordant at 989,747 of 1,003,031 SNP array positions (98.7% concordance).

### SNP and Indel annotation

We designed a pipeline to annotate SNPs and indels. The human gene annotation release 62 provided by Ensembl database ( http://www.ensembl.org/info/data/ftp/index.html) was used for annotating variants with gene, exon and UTRs. The repeat definition, conserved TFBS and enhancer information was obtained from UCSC genome browser database ( http://genome.ucsc.edu). SIFT annotation was performed using the online version available at ( http://sift.bii.a-star.edu.sg/). The pathway analysis was performed using DAVID program [35] and an FDR of < = 0.05 was used to identify significant pathways.

### Comparison and novel variants

The personal genome information was obtained from Ensembl, UCSC, Galaxy and published articles. The variant annotation for 1000 Genomes Project was obtained from ( http://www.1000genomes.org/). The common SNP database (dbSNP132) was downloaded from Ensembl and UCSC. Liftover program ( http://genome.ucsc.edu/cgi-bin/hgLiftOver) was used to convert the coordinate from hg18 to hg19 version of the genome.

### mtDNA analysis

From the comparison of the SAIF mt genome and the reference sequence (rCRS, NC_012920), 35 single nucleotide variants were found. Those variants were used to identify the haplotype of SAIF, using Haplogrep [95]. To examine phylogenetic relationships of the haplotype of SAIF with closely related haplotypes, the Neighbor-Joining tree was constructed by MEGA5 [96] for 210 complete mitochondrial genomes belonging to U and K haplogroups. The genome sequences were retrieved from GenBank. The coalescence time for the U haplogroups was estimated using BEAST [97]. For the BEAST analysis, 313 mt genome sequences evenly distributed throughout all lineages obtained from GenBank were used. The following parameters were used for the BEAST analysis: strict clock molecular clock model, exponential growth tree prior, Markov chain Monte Carlo (MCMC) chain length 2 M, and 10% burn-in.

### OMIM, SNPedia and varimed annotation

We compared the SNPs predicted from the SAIF genome against disease associated OMIM variants. We also annotated the SAIF genome against SNPedia to understand the effect of the variants. Annotation using Varimed database was performed as described recently [77]. Briefly, we first retrieved the SAIF’s genotypes, including variants and ref-ref calls for all the significant SNPs represented in the Varimed database known to be associated with disease based on genome-wide association studies. For multiple SNPs in the same linkage disequilibrium with R^2^ > 0.3, we only kept the one with the strongest evidence. Finally, we multiplied the likelihood ratio (LR) from independent SNPs, incorporated it with the pre-test probability to estimate the post-test probability of the disease.

## Competing interests

As noted some of the authors noted are employees of SciGenom Inc. SSe is an employee of Genentech and holds shares in Roche.

## Authors’ contributions

SSe, SCS, GT and SSa conceived the study. RG and RVD developed the algorithms for analysis of variants. RG performed the analysis and annotation of variants. AR, WM, and RB developed algorithms for variant and preformed the variant calling. AR and RG performed variant comparison with other personal genomes. RG, CR, SSe, and SSa performed the health relevant variant and pharmacogenomic analysis. HLK performed the mitochondrial DNA analysis. RC and AJB performed the Varimed analysis. SSe, SCS, AJB and GT provided oversight during the course of the study. RG, AR, CR, RC, HLK, SSe, and GT wrote and edited the manuscript. All authors read, edited and approved the manuscript.

## Data

Sequencing and genotype data has been deposited at the European Genome-Phenome Archive ( http://www.ebi.ac.uk/ega/), which is hosted by the EBI, under accession number EGAS00001000328. The SAIF variant information can be viewed at http://gbrowse.scigenom.com.

## Supplementary Material

Additional file 1 **Table S1.** SNPs and indels in (A) Gene, Regulatory and Enhancer regions, (B) Repeat class and family. Table S2 Non-synonymous SNPs in SAIF genome. Table S3 SNPs predicted to be damaging by SIFT. Table S4 (A) In-frame short indels, and (B) Short frameshift indels in SAIF genome. Table S5 Short indels predicted to lead to non-sense mediated decay (NMD) by SIFT. Table S6 SAIF SNP comparison. Table S7 Novel SNPs and indels in SAIF genome. Table S8 SAIF SNPs represented in OMIM. Table S9 SAIF SNPs annotated using SNPedia. Table S10 Pharmcogenomic relevant variants in SAIF genome. Click here for file

Additional file 2 **Figure S1.** Pathway analysis of synonymous SNPs. Pathway enrichment analysis was performed using DAVID program. The enriched KEGG pathways (FDR<= 0.05) identified are reported. **Figure S2.** Protein domain position and non-sense SNP location in MMP28 protein. **Figure S3.** Phylogenetic relationship of the SAIF mt genome. The tree on the left shows phylogenetic relationships of human mt macro-haplogroups. The right tree is a Neighbor-Joining tree of U and K haplogroups. The tree was constructed using 210 complete mt genome sequences, which were obtained from the GenBank database, including the SAIF mt genome (highlighted by red). The SAIF mitochondrial genome clustered with the U1 branch and was closely related to the U1a3 haplogroup. A comparison of the SAIF mt genomic sequence against the U1a3 sequence (GenBank accession # AY714038) revealed 14 nucleotide differences between the two genomes. **Figure S4.** Coalescence time estimations for the U haplogroup. The coalescence time for the U mt haplogroup was estimated by the BEAST analysis [97]. A total of 313 mt complete genome sequences obtained from GenBank that are representative of each macro-haplogroup and each U1 haplogroup were used in the analysis. We calibrated our time to most recent common ancestor (TMRCA) estimates based on published estimate of 660 kya for the separation of the *Homo sapiens* and *Neanderthal* mt lineages [98] and the 194.3 ± 32.55 kya TMRCA estimate for the global mtDNA genome tree [99]. BEAST analysis was run with HKY substitution model, the strict molecular clock model, exponential population growth tree prior, MCMC chain length 2M, and a 10% burn-in, as parameters. The coalescence time for the U haplogroup and U1a haplogroup were estimated to be 86 kya and 46 kya, respectively. **Figure S5.** Relative genetic risk of SAIF in comparison to GIH population represented in HapMap III. We used a set of disease SNPs measured in both SAIF and GIH, and recalculated the LR for SAIF and each of 101 GIH individuals. The histogram of the individual in each risk range is shown for each disease. SAIF individual had a higher genetic risk than 80% of GIH on eight diseases. **Figure S6.** Contribution of individual SNPs to the overall risk for uterine lyoma is shown. For explanation of the symbols and other parameters in the graph refer to Figure 8. **Figure S7.** Contribution of individual SNPs to the overall risk for asthma is shown. For explanation of the symbols and other parameters in the graph refer to Figure 8. **Figure S8.** Contribution of individual SNPs to the overall risk for obesity is shown. For explanation of the symbols and other parameters in the graph refer to Figure 8. Click here for file
