# Supplementary material for: Sequencing and analysis of a South Asian-Indian personal genome
Source: BMC Genomics. 2012 Aug 31;13:440. doi: 10.1186/1471-2164-13-440 (PMC3534380; doi:10.1186/1471-2164-13-440)
Supplement: Additional file 1 — Table S1. SNPs and indels in (A) Gene, Regulatory and Enhancer regions, (B) Repeat class and family. Table S2 Non-synonymous SNPs in SAIF genome. Table S3 SNPs predicted to be damaging by SIFT. Table S4 (A) In-frame short indels, and (B) Short frameshift indels in SAIF genome. Table S5 Short indels predicted to lead to non-sense mediated decay (NMD) by SIFT. Table S6 SAIF SNP comparison. Table S7 Novel SNPs and indels in SAIF genome. Table S8 SAIF SNPs represented in OMIM. Table S9 SAIF SNPs annotated using SNPedia. Table S10 Pharmcogenomic relevant variants in SAIF genome. [file 1471-2164-13-440-S1.pdf]

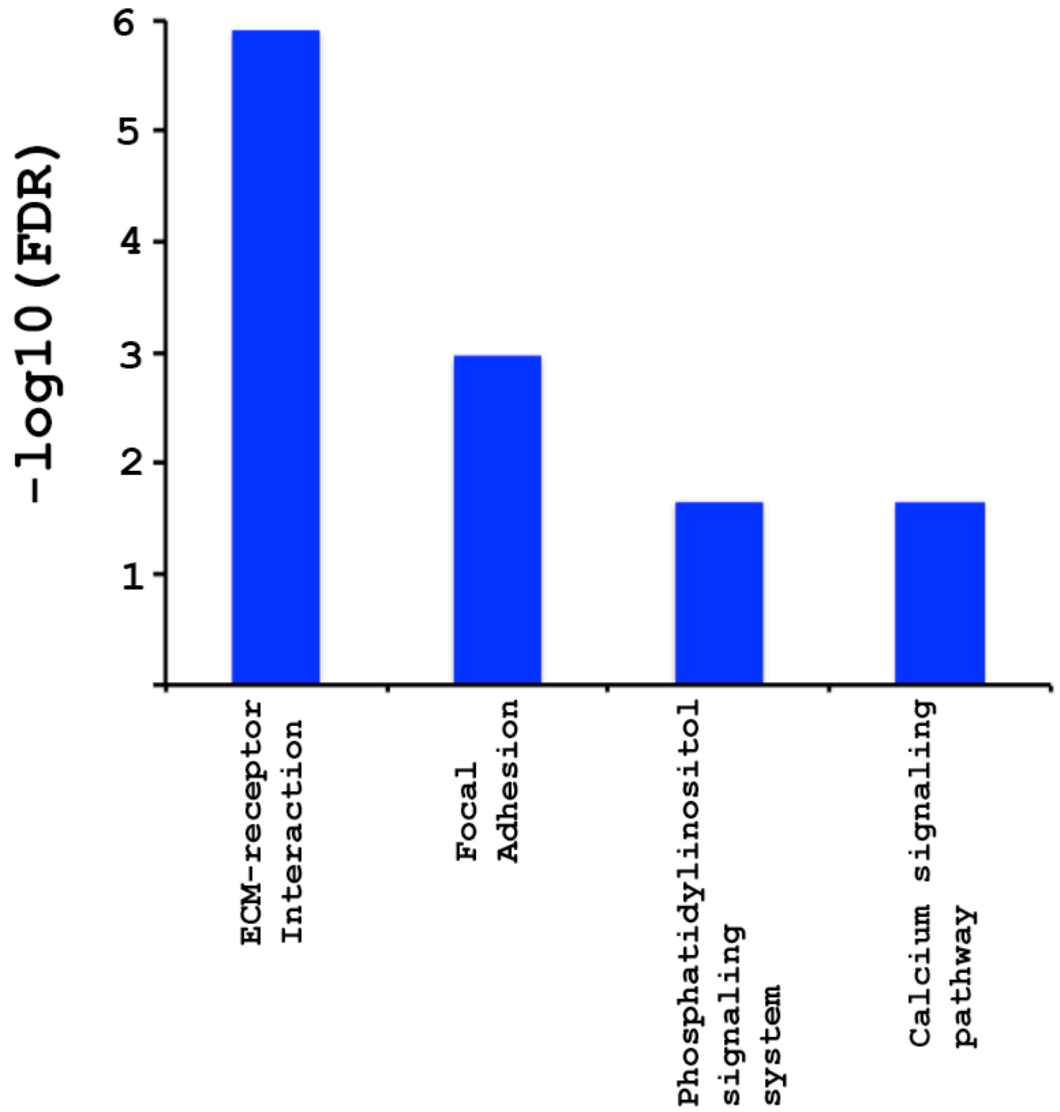

Supplementary Fig. 1

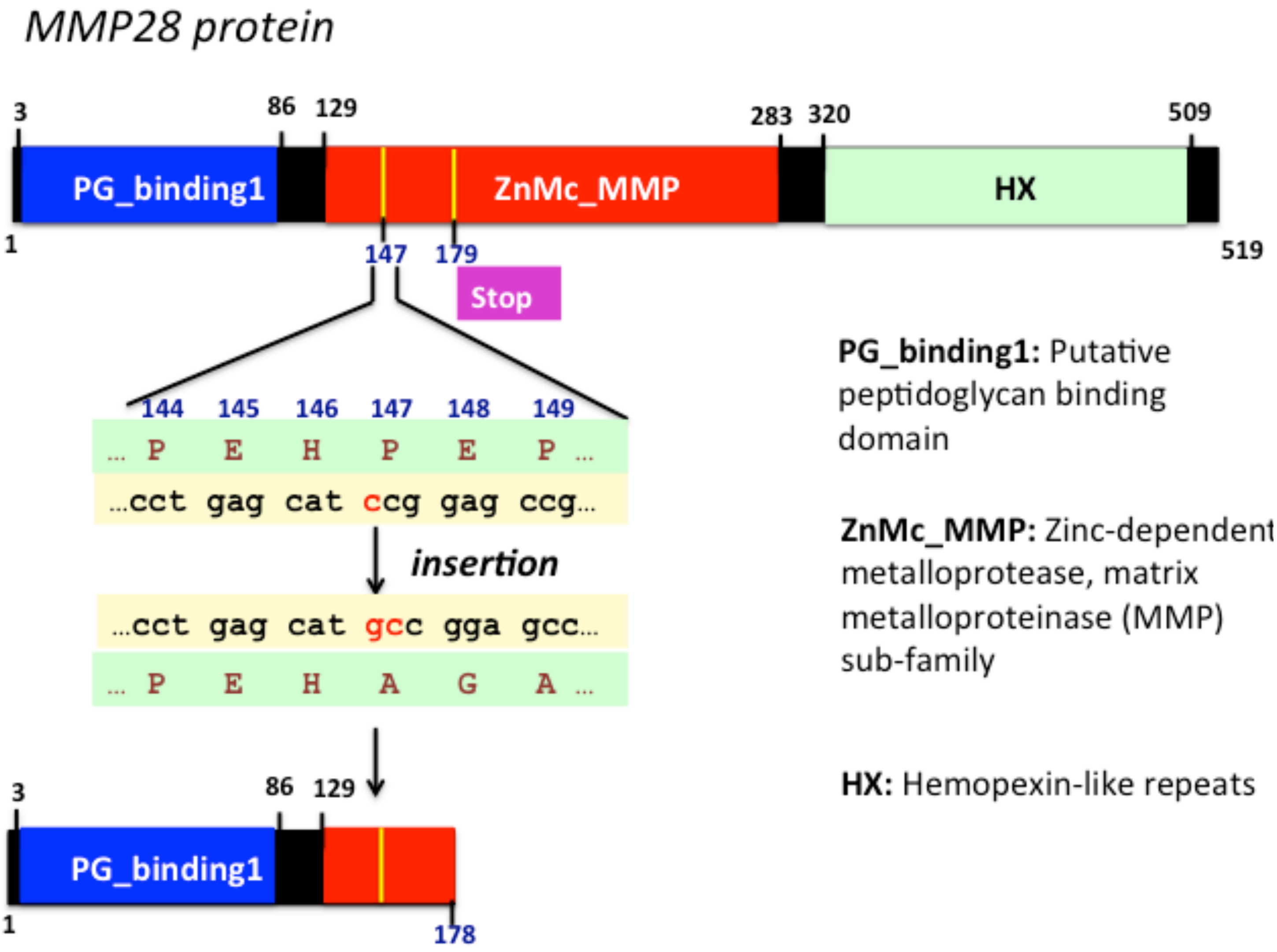

Supplementary Fig. 2

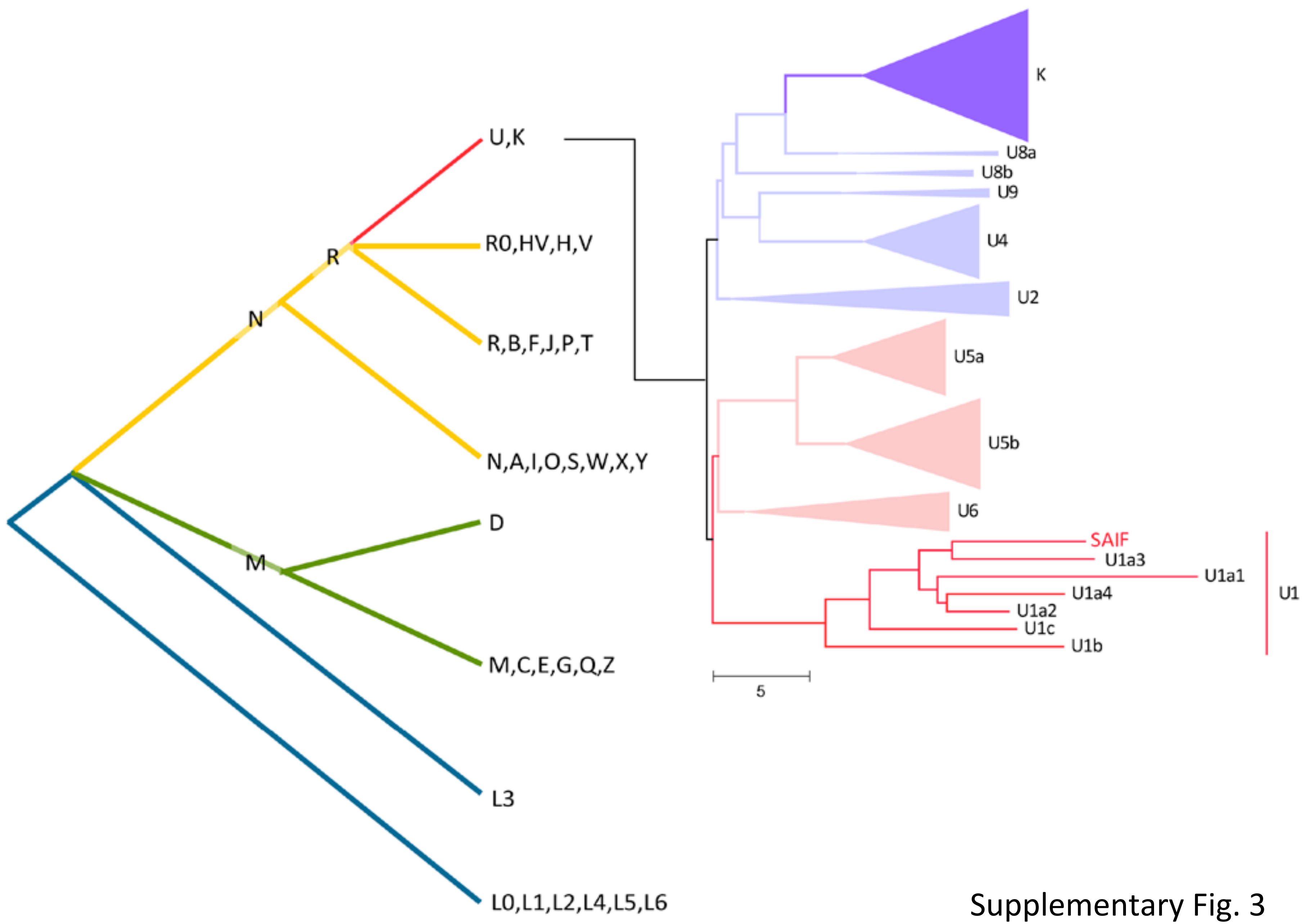

Supplementary Fig. 3

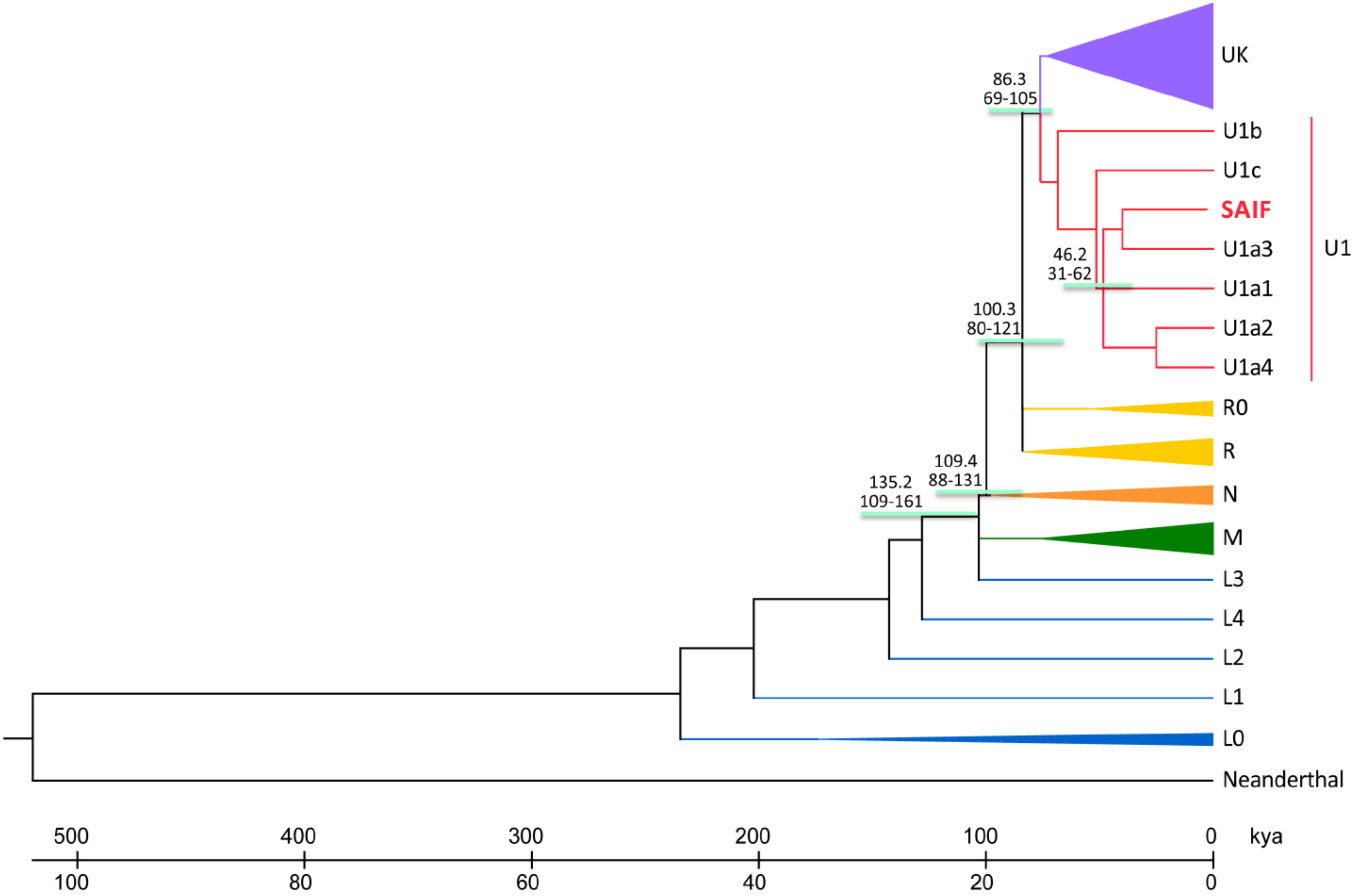

Supplementary Fig. 4

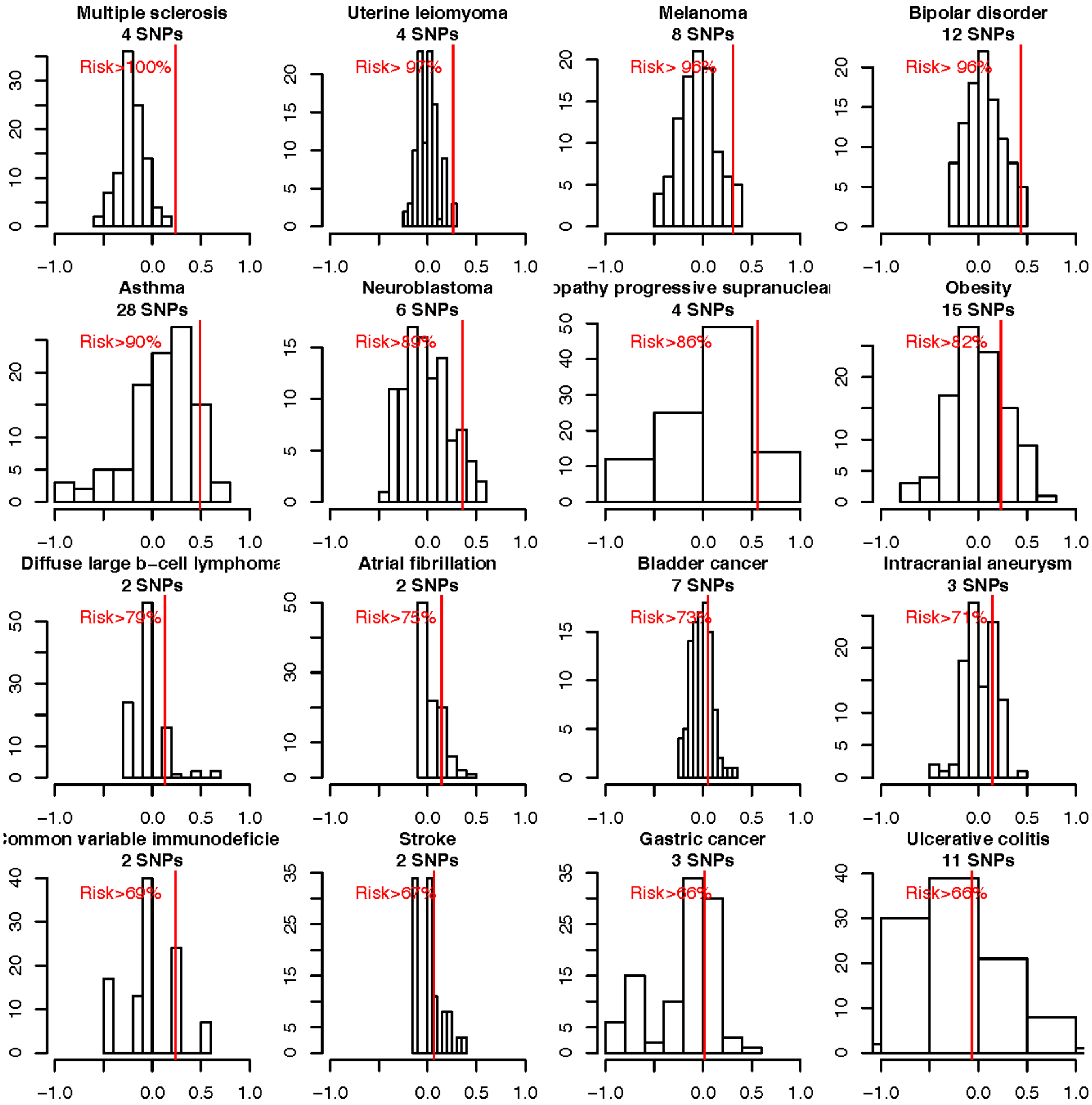

Supplementary Fig. 5

Uterine leiomyoma

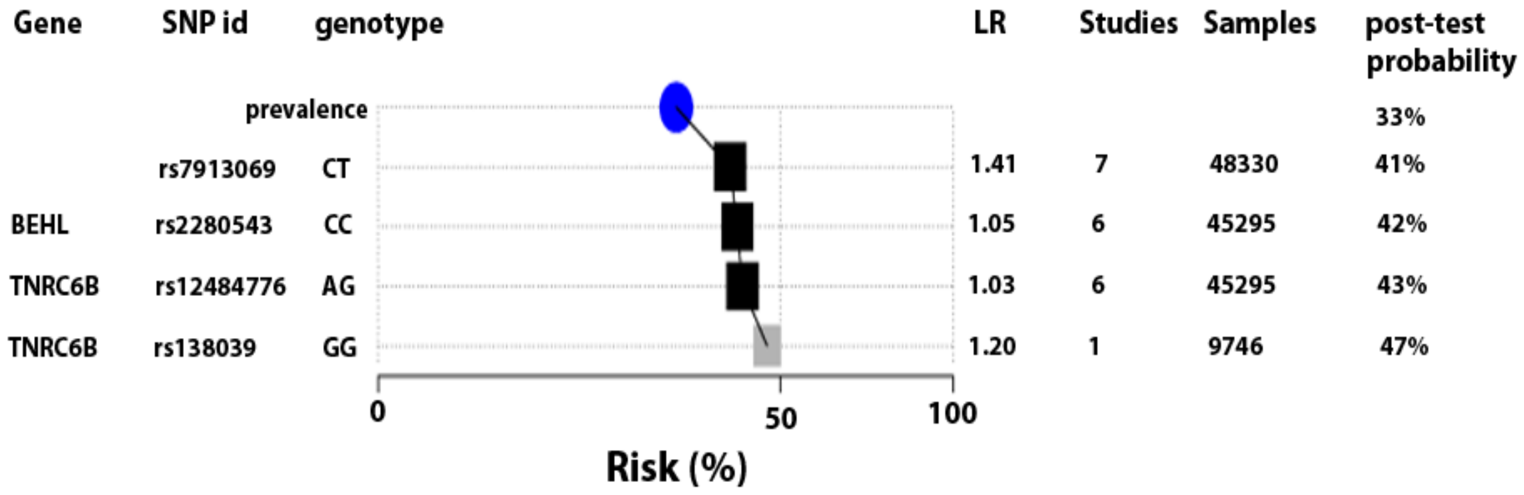

Supplementary Fig. 6

Asthma

Gene	SNP Id	Genotype	LR	Studies	Samples	Post-test Probability
Prevalance						13%
GSDMA	rs3894194	GG	0.80	6	34622	11%
	rs1837253	CC	1.17	4	65330	12%
DENND1B	rs1924518	GG	1.05	4	11737	13%
	rs1701704	TT	0.93	3	70166	12%
PBX2	rs204993	AA	1.14	3	70166	13%
	rs412657	TT	1.22	3	70166	16%
IL18R1	rs3771166	GA	1.03	3	34622	16%
	rs1342326	AC	0.86	3	34622	15%
DENND1B	rs2111931	TT	1.11	3	8025	16%
CRB1	rs2786098	GG	1.11	3	8025	17%
PDE4D	rs1588265	AA	1.36	3	1205	22%
IL1RL1	rs1420101	CT	1.04	2	102962	23%
	rs10508372	GA	0.99	2	65330	23%
	rs7686660	TT	1.18	2	65330	26%
NOTCH4	rs404860	TT	1.19	2	65330	29%
C6orf10	rs570963	AA	1.14	2	65330	32%
	rs3117098	GA	1.09	2	65330	34%
	rs3129890	TT	1.10	2	65330	36%
C6orf10	rs3129943	AA	1.11	2	65330	38%
	rs7775228	TT	1.11	2	65330	41%
	rs9275698	AA	1.05	2	65330	42%
BTNL2	rs10947262	CC	1.11	2	65330	45%
C6orf10	rs10484560	GG	0.92	2	39919	43%
IL13	rs1295686	CC	0.78	2	34622	37%
	rs9273349	TT	0.85	2	25735	33%
SMAD3	rs744910	GA	0.99	2	20119	33%
RORA	rs11071559	CC	1.32	2	20119	40%
IL2RB	rs2284033	GA	1.01	2	20119	40%
GAB1	rs3805236	GG	1.14	1	35083	43%
NOTCH4	rs443198	AG	1.00	1	35083	43%
	rs3130299	AA	0.93	1	35083	41%
BTNL2	rs3806156	GG	1.11	1	35083	44%
	rs6457617	CC	0.85	1	35083	40%
	rs9500927	GG	0.93	1	35083	38%
SLC22A5	rs2073643	TT	1.10	1	20119	40%
	rs1882766	CT	0.98	1	4836	40%

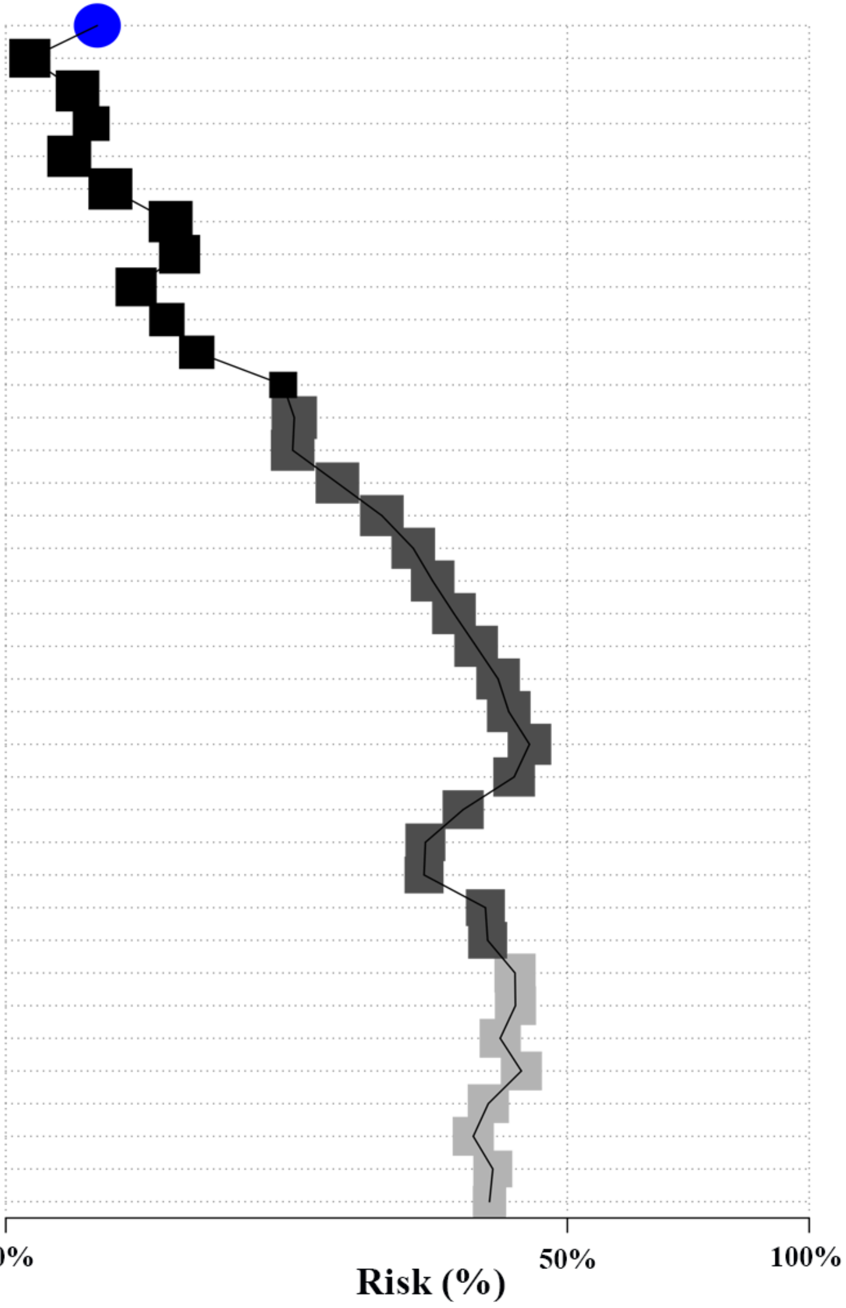

Supplementary Fig. 7

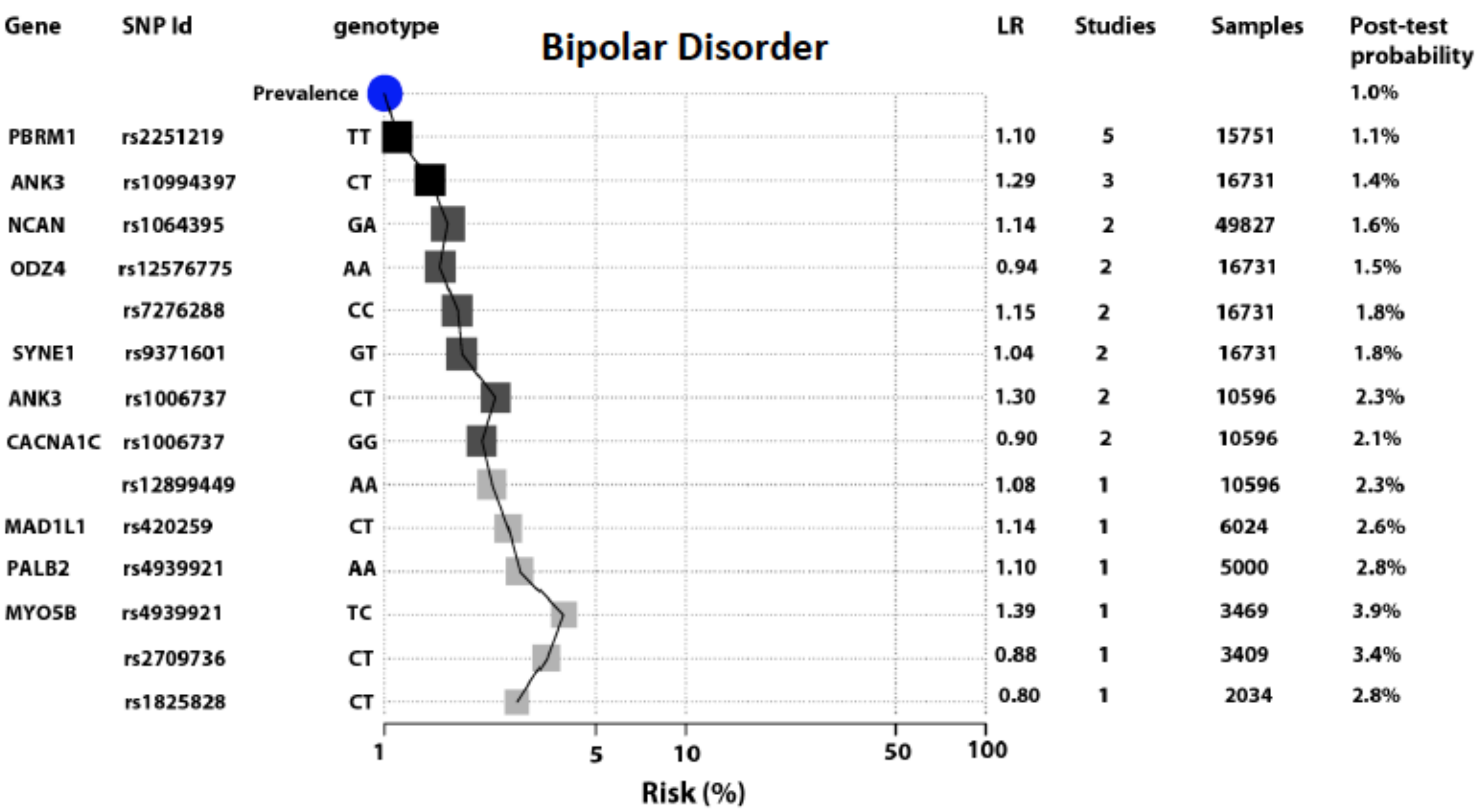

Supplementary Fig. 8
